# *Kudoa septempunctata* Parasite–Associated Foodborne Disease Outbreaks, South Korea, 2015–2024

**DOI:** 10.3201/eid3208.260653

**Published:** 2026-08

**Authors:** Seongdae Kim, Byung Chul Chun

**Affiliations:** Hongik University, Seoul, South Korea (S. Kim); Korea University Graduate School, Seoul (S. Kim, B.C. Chun); Korea University College of Medicine, Seoul (B.C. Chun)

**Keywords:** Kudoa septempunctata, parasites, food safety, foodborne disease, olive flounder, interrupted time series, COVID-19, South Korea, raw fish, surveillance

## Abstract

We analyzed 2015–2024 national surveillance data on *Kudoa septempunctata* parasite–associated foodborne disease in South Korea. Monthly cases decreased 95% during the COVID-19 pandemic, with no significant recovery through 2024; affected municipalities dropped from 89 to 27. Our findings are consistent with restaurant-based raw fish consumption as the principal transmission pathway.

*Kudoa septempunctata* is a myxosporean parasite that causes transient gastroenteritis within hours of consuming raw infected fish (*1*). Although best known from farmed olive flounder (*Paralichthys olivaceus*), it has been reported from phylogenetically diverse marine fishes, including members of the order Tetraodontiformes (black scraper [*Thamnaconus modestus*], wild grass puffer [*Takifugu alboplumbeus*]) and the series Eupercaria (Japanese whiting [*Sillago japonica*]), indicating that human exposure is not limited to olive flounder (*2*). After the *K. septempunctata* parasite was identified in Japan in 2011 (*1*), South Korea, the world’s largest producer of farmed olive flounder, designated it a notifiable foodborne pathogen in 2015. However, national surveillance during the COVID-19 pandemic has not been analyzed. We examined whether pandemic-related restrictions on dining out interrupted *K. septempunctata* parasite incidence and whether a rebound followed restoration of normal dining behaviors, thereby assessing whether restaurant-based exposure is the dominant transmission pathway.

We obtained monthly outbreak and case data for 2015–2024 from the Ministry of Food and Drug Safety surveillance system, aggregated by municipality (si-gun-gu, which consists of cities, counties, and districts; n = 229) and linked to Statistics Korea population data. Using February 2020 as the intervention point, we fitted a negative binomial interrupted time series model with a population offset and Fourier seasonality terms (K = 2). We compared municipality-level choropleth maps of crude incidence rates and Getis–Ord statistic hotspots between the pre–COVID-19 (2015–2019) and COVID-19/postpandemic (2020–2024) periods (Appendix Figure 1). 

During 2015–2024, a total of 1,230 cases and 215 *K. septempunctata* outbreaks were reported (Table). Prepandemic, annual cases nearly tripled from 114 in 2015 to 308 in 2019, and outbreaks increased from 15 to 48. Restaurants accounted for 167 (77.7%) of all outbreaks, and no household outbreak was reported in any year, implicating commercial food-service venues as the dominant exposure setting (*3*). Cases dropped abruptly to 40 in 2020 and 8 in 2021, then increased gradually to 70 in 2024.

**Table:** Characteristics of cases and settings for *Kudoa septempunctata* parasite–associated foodborne disease outbreaks, South Korea, 2015–2024*

Year	No. outbreaks	No. cases	No. cases/outbreak	Incidence rate, cases/100,000 population	Outbreak setting, no. (%)	
					Restaurant	Other facility†
Pre–COVID-19 period						
2015	15	114	7.6	0.22	11 (73.3)	4 (26.7)
2016	39	212	5.4	0.41	32 (82.1)	7 (17.9)
2017	39	177	4.5	0.34	31 (79.5)	8 (20.5)
2018	37	229	6.2	0.44	25 (67.6)	12 (32.4)
2019	48	308	6.4	0.59	36 (75.0)	12 (25.0)
COVID-19 and postpandemic period						
2020	10	40	4.0	0.08	10 (100.0)	0
2021	2	8	4.0	0.02	2 (100.0)	0
2022	6	24	4.0	0.05	5 (83.3)	1 (16.7)
2023	11	48	4.4	0.09	9 (81.8)	2 (18.2)
2024	8	70	8.8	0.14	6 (75.0)	2 (25.0)
Total	215	1,230	5.7	0.24	167 (77.7)	48 (22.3)

*Total incidence rate represents the annual average calculated as total cases divided by mean annual population over the 10-y study period. Outbreak and setting data, Ministry of Food and Drug Safety surveillance system; population data, Statistics Korea. No home-setting outbreaks were reported throughout the study period. Pre–COVID-19 period January 2015–February 2020; COVID-19 and postpandemic period March 2020–December 2024. †Encompasses food service settings not classified as schools, group catering facilities, or private residences, including convenience stores, retail markets, and street food vendors.

The interrupted time series analysis (Figure) showed a highly significant reduction at COVID-19 onset (β_2_ = −2.948 [95% CI, −3.919 to −1.989]; p<0.001), corresponding to a 95% drop in monthly cases. The postpandemic slope showed no significant recovery (Appendix Table 1). Affected municipalities contracted from 89 (38.9%) to 27 (11.8%). Before the pandemic, incidence was highest along coastal municipalities and Jeju Island, and Getis–Ord statistic hotspots clustered in the Incheon area; afterward, incidence concentrated along the Gangwon Province east coast, a major raw fish dining destination (Appendix Figure 1).

**Figure:**
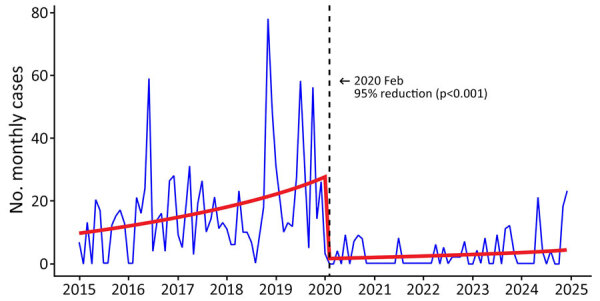
Interrupted time series analysis of monthly *Kudoa septempunctata* parasite–associated foodborne disease cases, South Korea, January 2015–December 2024. Blue line, observed monthly counts; red line, model-fitted trend with seasonality removed; dashed vertical line, COVID-19 intervention point (February 2020). The model was a negative binomial regression with a population offset and Fourier terms (K = 2). The level change at COVID-19 onset corresponds to a 95% reduction in monthly cases (β_2_ = –2.948 [95% CI –3.919 to –1.989]; p<0.001).

The pandemic abruptly altered raw fish consumption, food distribution, travel, and dining behaviors, providing an opportunity to observe changes in the epidemiology of this obligate raw fish–borne pathogen. *K. septempunctata* outbreaks were almost exclusively restaurant-based, and no household-associated outbreaks were reported, likely reflecting both the concentration of raw flounder consumption in commercial venues and the greater ease of detecting and tracing clustered restaurant exposures than dispersed household cases. Therefore, restrictions on dining out directly interrupted the dominant exposure pathway. South Korea’s COVID-19 response was distinctively rigorous, including legally enforced restaurant capacity, operating hours, and group size limits, with administrative penalties for noncompliance (*4*); South Korea foodservice spending contracted by 12% in 2020 and continued a sustained decline through 2021 (data via Statistics Korea), removing the venues from concentrating *K. septempunctata* exposure. A parallel decline in *K. septempunctata* outbreaks was reported from Japan (*5*).

The absence of a postpandemic increase in outbreaks contrasts with, rather than parallels, broader patterns for person-to-person enteric pathogens. Whereas norovirus and similar pathogens rebounded after nonpharmaceutical interventions ended through renewed human-to-human transmission (*6*), *K. septempunctata* parasites have no human-to-human transmission, depending entirely on a specific food vehicle; therefore, the transmission chain plausibly remained disrupted at multiple points. Mandatory inspection protocols for South Korea olive flounder farms after 2015 likely reduced contamination at the source (*7*), whereas enhanced molecular diagnostics (*8*) and durable consumer shifts toward delivery and home cooking (*9*) further constrained transmission. The gradual increase in cases during 2023–2024 nonetheless signals possible reemergence as raw fish dining increases.

Limitations of our study include possible reporting disruption during the pandemic, which could inflate the apparent decline, and likely underascertainment given transient symptoms and limited diagnostic availability (*10*). The single-group interrupted time series design precludes counterfactual inference, and concurrent changes in aquaculture inspection and dining behavior could not be disentangled.

In conclusion, the COVID-19 pandemic coincided with interruption of an emerging *K. septempunctata* outbreak trend in South Korea and a sustained decline through 2024. The convergence of temporal, spatial, and setting-level evidence is consistent with restaurant-based raw fish consumption being the principal transmission pathway, although the observational, single-group design and multiple concurrent changes preclude definitive causal attribution. For an international readership, our findings show how abrupt behavior change can reveal a food-specific parasite’s dominant exposure setting and how aquaculture source-control could sustain reductions. We call for renewed surveillance during 2025–2026 as rates of raw fish dining normalize toward prepandemic levels and for integrating consumer behavior monitoring with aquaculture inspection in coastal high-risk areas.

Appendix Additional information for *Kudoa septempunctata* parasite–associated foodborne disease outbreaks, South Korea, 2015–2024.
