# Supplementary material for: Kudoa septempunctata Parasite–Associated Foodborne Disease Outbreaks, South Korea, 2015–2024
Source: Emerg Infect Dis. 2026 Aug;32(8):1373–5. doi: 10.3201/eid3208.260653 (PMC13426883; doi:10.3201/eid3208.260653)
Supplement: Appendix — Additional information for Kudoa septempunctata parasite–associated foodborne disease outbreaks, South Korea, 2015–2024. [file 26-0653-Techapp-s1.pdf]

Article DOI: https://doi.org/10.3201/eid3208.260653

EID cannot ensure accessibility for supplementary materials supplied by authors. Readers who have difficulty accessing supplementary content should contact the authors for assistance.

Kudoa septempunctata Parasite–Associated Foodborne Disease Outbreaks, South Korea, 2015–2024

Appendix

Host Range and Food-Safety Context

Although Kudoa septempunctata is best known as a parasite of farmed olive flounder (Paralichthys olivaceus), recent evidence shows that its host range extends well beyond this single species. The review by Shamsi and Barton (*1*) reports that K. septempunctata has been recorded from phylogenetically diverse marine fishes, including members of the order Tetraodontiformes—such as the black scraper (Thamnaconus modestus, family Monacanthidae) and the wild grass puffer (Takifugu alboplumbeus, family Tetraodontidae)—and of the series Eupercaria, such as the Japanese whiting (Sillago japonica, family Sillaginidae). Two congeneric species, K. septempunctata and K. hexapunctata, have been implicated in human gastrointestinal illness, and new hosts and expanded geographic distributions continue to be described. For food-safety surveillance, this broader and apparently expanding host range implies that human exposure is not necessarily confined to olive flounder; raw or undercooked consumption of other susceptible species could, in principle, contribute to sporadic or unrecognized illness. Diagnosis remains challenging because symptoms are transient and nonspecific, molecular confirmation (18S rDNA PCR) is available mainly in specialized laboratories, and routine inspection has historically focused on olive flounder. These considerations reinforce the value of species-aware aquaculture inspection, extended molecular surveillance capacity, and interpreting national case counts as a likely underestimate of the true burden. In the present study, all reported outbreaks were linked to olive flounder, consistent with its dominance in the Korean raw-fish market; the wider host range is presented here as context for the parasite's emerging food-safety relevance rather than as a feature of the surveillance data analyzed.

Materials and Methods

Data Sources and Preparation

Monthly Kudoa septempunctata–associated foodborne disease case counts and outbreak setting data (January 2015–December 2024) were obtained from the Ministry of Food and Drug Safety (MFDS) foodborne disease surveillance system, which compiles laboratory-confirmed cases under the Food Sanitation Act through nationwide notification and outbreak-investigation channels. Population denominators (mid-year resident population) were obtained from Statistics Korea (KOSIS). Both data sources operate as nationwide population-based reporting systems with stable case definitions throughout the study window. Cases and outbreaks were aggregated at the municipality (si/gun/gu) level, yielding 229 spatial units. Two administrative-boundary corrections were applied to harmonize the surveillance data with the geographic shapefile (final.shp, EPSG 5179 → 4326 reprojected to WGS-84): (i) Incheon Michuhol-gu observations recorded under its prior name (Nam-gu) were re-coded to the current Michuhol-gu unit; (ii) Gunwi-gun observations recorded under Gyeongsangbuk-do were re-coded to its current administrative parent (Daegu Metropolitan City), reflecting the 2023 administrative reassignment. Annual case totals were verified against published MFDS annual reports; one minor correction (308 cases for 2019, replacing an aggregation rounding artifact of 302) was applied to reconcile the surveillance database with the published annual figure.

Ethics

This study was approved by the Institutional Review Board of Hongik University (approval no. 7002340-202511-HR-012). Informed consent was waived because only de-identified, aggregated municipal-level surveillance data were analyzed.

Interrupted Time Series Model

We fitted a negative binomial generalized linear model (function MASS::glm.nb in R) to monthly national case counts, following the segmented interrupted time series approach for evaluating public health interventions (*2*), with municipal mid-year population as a log-offset to model rates rather than counts. The model was specified as $\log(E[Y_t]) = \log(pop_t) + \beta_0 + \beta_1 \cdot time_t + \beta_2 \cdot COVID_t + \beta_3 \cdot time\_after_t + \Sigma_{k = 1..2}[\alpha_k \cdot \sin(2\pi k \cdot t/12) + \gamma_k \cdot \cos(2\pi k \cdot t/12)]$, where Y_t denotes the observed monthly case count in calendar month t; pop_t is the corresponding national mid-year population (entered as offset(log(pop))); $time_t$

(= 1, 2, …, 120) is a monthly running counter (January 2015 = 1; December 2024 = 120); COVID_t is the level-change indicator (0 for t < February 2020 and 1 thereafter); time_after_t is the post-intervention monthly counter (counting from February 2020 = 1, otherwise 0); and the Fourier sine and cosine pairs ($\sin(2\pi \cdot t/12)$, $\cos(2\pi \cdot t/12)$, $\sin(4\pi \cdot t/12)$, $\cos(4\pi \cdot t/12)$) capture annual and semi-annual seasonality at harmonic order $K = 2$. The negative binomial distribution was chosen over Poisson to accommodate over-dispersion characteristic of count surveillance time-series. Fourier terms were generated with forecast::fourier() applied to the ts() object (*3*). The intervention point of February 2020 corresponds to the first national social-distancing measures in South Korea following the Daegu COVID-19 cluster.

Coefficient Interpretation

The pre-COVID-19 monthly trend β_1 captures the rate of change in log-cases before the intervention, and $\exp(\beta_1)$ gives the monthly multiplicative change in expected cases (per population). The level change β_2 at COVID-19 onset represents the immediate step change at February 2020; $\exp(\beta_2)$ gives the multiplicative change in expected monthly cases at the intervention, with $\exp(-2.948) \approx 0.052$ corresponding to a $1 - 0.052 = 94.8\%$ (rounded to 95%) reduction. The post-COVID-19 trend change β_3 represents the additional monthly slope in the post-intervention period, so the post-COVID monthly trend equals $\beta_1 + \beta_3$; β_3 near zero with non-significant p indicates that the post-pandemic slope is not statistically distinguishable from the pre-pandemic slope, but the level itself remains depressed by $\exp(\beta_2)$.

Residual Diagnostics

Model adequacy was assessed by inspection of Pearson residuals and a Ljung–Box test (Box.test(..., lag = 12, type = “Ljung-Box”)) on these residuals. The test yielded $p = 0.458$, indicating no statistically significant residual autocorrelation up to lag 12 months and supporting the appropriateness of the Fourier-$K = 2$ seasonality specification.

Spatial Analysis: Choropleth Maps

Crude incidence rates (cases per 100,000 population) were computed for each municipality for two periods: pre-COVID-19 (2015–2019) and COVID-19 / post-pandemic (2020–2024). The denominator for each period was the period-mean municipal population. Maps used a five-category classification: 0 (no reported cases) shown in white; Q1–Q4 representing the empirical quartiles of the strictly positive period-incidence distribution.

Spatial Analysis: Getis-Ord Gi* Hotspot Analysis

Local spatial association was assessed with the Getis-Ord Gi* statistic (*4*) (spdep::localG_perm(), R package spdep). Spatial weights were defined by queen contiguity (sharing an edge or vertex), row-standardized. Statistical significance was assessed by 999 Monte Carlo permutations. Maps display the resulting Z-scores in seven categories: >2.58 (high-incidence hotspot, $p < 0.01$); 1.96 to 2.58 (high-incidence hotspot, $p < 0.05$); 1.65 to 1.96 (high-incidence hotspot, $p < 0.10$); −1.65 to 1.65 (not significant); −1.96 to −1.65 (low-incidence cold spot, $p < 0.10$); −2.58 to −1.96 (low-incidence cold spot, $p < 0.05$); <−2.58 (low-incidence cold spot, $p < 0.01$). No statistically significant cold spots were identified in either period, reflecting the predominance of zero-incidence municipalities (n = 140 of 229 in 2015–2019 and n = 202 of 229 in 2020–2024).

Note on Seasonality

Inspection of the monthly case time series revealed a bimodal seasonal pattern with peaks in April and October–November. The October peak aligns with the seasonal peak previously reported for K. septempunctata food poisoning in Japan (*5*). Because *K. septempunctata* spores are detected in olive flounder year-round and do not exhibit a clear water-temperature dependency, the observed seasonality is more plausibly attributable to seasonal variation in raw-fish consumption (April: spring outdoor-dining onset; October–November: autumn fishing and travel season) than to environmental amplification of the parasite. This bimodal pattern was statistically controlled for in the ITS model via the Fourier K = 2 seasonality terms; we present the seasonal observation here for descriptive completeness rather than as a hypothesis test.

Statistical Software

All analyses were performed in R version 4.6.0. Key packages: MASS (negative binomial regression), forecast (Fourier seasonal terms), sf and spdep (spatial weights and Gi* statistic), ggplot2 and patchwork (figures), openxlsx (table export), dplyr/tidyr (data manipulation).

References

1. Shamsi S, Barton DP. Exploring the potential role of the genus Kudoa (Myxosporea: Kudoidae) as an emerging seafood-borne parasite in humans. Curr Clin Microbiol Rep. 2024;11:107–14. https://doi.org/10.1007/s40588-024-00220-1

2. Lopez Bernal J, Cummins S, Gasparrini A. Interrupted time series regression for the evaluation of public health interventions: a tutorial. Int J Epidemiol. 2017;46:348–55. PubMed https://doi.org/10.1093/ije/dyw098

3. Hyndman RJ, Khandakar Y. Automatic time series forecasting: the forecast package for R. J Stat Softw. 2008;27:1–22. https://doi.org/10.18637/jss.v027.i03

4. Getis A, Ord JK. The analysis of spatial association by use of distance statistics. Geogr Anal. 1992;24:189–206. https://doi.org/10.1111/j.1538-4632.1992.tb00261.x

5. Hadano Y, Mori H, Tanaka Y, Mahittikorn A, Ohno S. Epidemiology of Kudoa septempunctata food poisoning in Japan from 2013 to 2023. Sci Rep. 2026;16:7986. PubMed https://doi.org/10.1038/s41598-026-38632-2

Appendix Table. Interrupted time series analysis of Kudoa septempunctata–associated foodborne disease, South Korea, 2015–2024.

	Pre-COVID-19 trend (*β1*)		COVID-19 level change (*β2*)		Post-COVID-19 trend (*β3*)		Ljung-Box
Disease	Est (95% CI)	*p*	Est (95% CI)	*p*	Est (95% CI)	*p*	*p*
K. septempunctata	0.0173 (−0.0011, 0.0357)	0.054	−2.9480 (−3.9194, −1.9891)	<0.001	0.0015 (−0.0248, 0.0279)	0.9140	0.458

$β_1$, pre-COVID-19monthly trend (slope); $β_2$, level change at COVID-19 onset (February 2020); $β_3$, change in post-COVID-19 trend (slope change). The negative binomial generalized linear model included a population offset and Fourier seasonality terms (K = 2). Ljung–Box p > 0.05 indicates no significant residual autocorrelation in the Pearson residuals (lag 12). Est, estimate; CI, confidence interval. Effect-size translation: exp($β_2$) = 0.0526, corresponding to a 94.74% reduction in expected monthly cases at COVID-19 onset (rounded to 95% in the main text); 95% CI bounds translate to a 98.0% reduction (exp(−3.919) = 0.0198) at the lower bound and an 86.3% reduction (exp(−1.989) = 0.1369) at the upper bound. exp($β_1$) = 1.0175 corresponds to a ~1.75% multiplicative monthly increase pre-pandemic (~23% per year). exp($β_3$) ≈ 1.00 indicates no detectable change in slope post-pandemic.

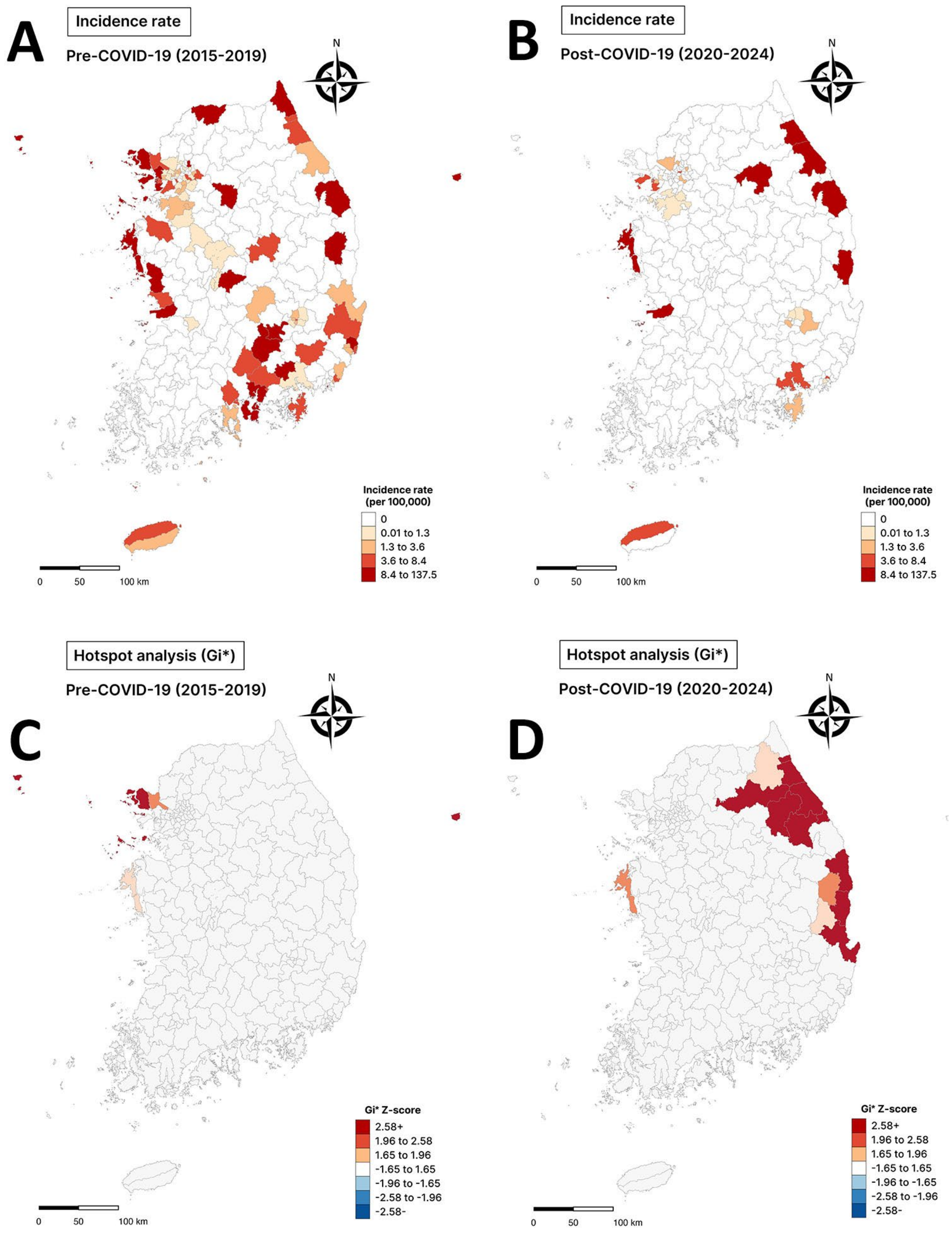

Appendix Figure. Municipal-level (si/gun/gu; n = 229) spatial distribution of Kudoa septempunctata–associated foodborne disease, Republic of Korea. A) Pre-COVID-19 period (2015–2019) crude incidence rate per 100,000 population, displayed on a five-category quartile classification with zero-incidence municipalities shown in white. B) COVID-19 / post-pandemic period (2020–2024), same classification scheme as (A). C) Pre-COVID-19 Getis-Ord Gi* hotspot map of crude incidence rates; Z-score classification with significance thresholds at |z| = 1.65, 1.96, and 2.58 (queen contiguity, row-

standardized; 999 Monte Carlo permutations). Red shading denotes high-incidence hotspots; blue shading would denote low-incidence cold spots, but none were identified at the chosen significance thresholds. D) COVID-19 / post-pandemic Getis-Ord Gi* hotspot map, same specification as (C). During the pre-pandemic period (panel A), 89 of 229 municipalities (38.9%) reported one or more cases, with the highest incidence rates concentrated along coastal municipalities (Incheon Metropolitan area on the western coast; Gangwon Province on the eastern coast) and Jeju Island (the center of olive flounder aquaculture); the corresponding hotspot panel (C) identified a statistically significant high-incidence cluster in the northwestern coastal and island areas (Incheon area), with an isolated significant signal on the eastern offshore island of Ulleung. During the COVID-19 / post-pandemic period (panels B and D), the number of municipalities reporting any case contracted to 27 (11.8%), and the hotspot pattern shifted to predominant concentration along the Gangwon Province east coast, a major raw-fish dining destination.
